# Evolution of a fatty acyl–CoA elongase underlies desert adaptation in *Drosophila*

**DOI:** 10.1126/sciadv.adg0328

**Published:** 2023-08-30

**Authors:** Zinan Wang, Jian Pu, Cole Richards, Elaina Giannetti, Haosu Cong, Zhenguo Lin, Henry Chung

**Affiliations:** ^1^ Department of Entomology, Michigan State University, East Lansing, MI 48824, USA.; ^2^ Ecology, Evolution, and Behavior Program, Michigan State University, East Lansing, MI 48824, USA.; ^3^ College of Agriculture, Sichuan Agricultural University, Chengdu, Sichuan 611130, China.; ^4^ Department of Biology, Saint Louis University, St. Louis, MO 63104, USA.

## Abstract

Traits that allow species to survive in extreme environments such as hot-arid deserts have independently evolved in multiple taxa. However, the genetic and evolutionary mechanisms underlying these traits have thus far not been elucidated. Here, we show that *Drosophila mojavensis*, a desert-adapted fruit fly species, has evolved high desiccation resistance by producing long-chain methyl-branched cuticular hydrocarbons (mbCHCs) that contribute to a cuticular lipid layer reducing water loss. We show that the ability to synthesize these longer mbCHCs is due to evolutionary changes in a fatty acyl–CoA elongase (*mElo*). *mElo* knockout in *D. mojavensis* led to loss of longer mbCHCs and reduction of desiccation resistance at high temperatures but did not affect mortality at either high temperatures or desiccating conditions individually. Phylogenetic analysis showed that *mElo* is a *Drosophila*-specific gene, suggesting that while the physiological mechanisms underlying desert adaptation may be similar between species, the genes involved in these mechanisms may be species or lineage specific.

## Teaser

Desert adaptation in Drosophila is due to evolutionary changes in a single gene.

## INTRODUCTION

The divergence and evolution of adaptive traits allow organisms to survive and thrive in diverse and extreme environments (*1*–*3*). A key feature of these extreme environments is having multiple abiotic factors of which the levels are beyond the physiologically tolerable ranges of most species (*4*). In many cases, the interaction between abiotic factors may exacerbate the stresses caused by these factors to organisms that live in the environments (*5*–*7*). For example, in hot-arid deserts, the increased organismal water loss due to the extremely low moisture content in the air is exacerbated by high temperatures, leading to even more rapid water loss (*8*, *9*). Nevertheless, species that are able to survive in these environments have evolved traits that allow them to withstand these stresses.

To survive rapid water loss in deserts, species from different taxa evolved high levels of desiccation resistance via similar physiological changes such as reducing water evaporation from the body, lowering metabolism, and minimizing water excretion (*10*–*12*). While there are some studies on these independently evolved physiological traits (*13*–*15*), the underlying molecular and evolutionary mechanisms remain largely unknown. Recent association studies using genomic and transcriptomic studies have identified some candidate genes that may contribute to physiological adaptation in desert organisms (*16*–*18*), but the functions of these genes are not characterized. In addition, it is not clear whether these adaptive traits in diverse desert species share the same underlying genetic mechanisms or are specific to different lineages of species. Determining the genetic basis underlying the evolution of desert adaptative traits may allow the prediction of whether and how contemporary species will evolve and adapt to future environmental changes such as global desertification (*19*, *20*).

Insects synthesize a hydrophobic lipid layer of cuticular hydrocarbons (CHCs) on their body surfaces as a major physiological mechanism to reduce cuticular water loss and resist desiccation (*13*, *21*, *22*). The chemical composition of this CHC layer, which is made up of different types of hydrocarbons such as *n*-alkanes, monoenes, dienes, and methyl-branched alkanes (or mbCHCs), is responsible for its biophysical properties, including its melting temperature and ability to reduce water loss (*23*, *24*). Comparative studies across several insect lineages showed a positive association between having longer CHCs and living in habitats with higher temperatures and lower precipitation (*25*–*27*). In *Drosophila*, experimental evidence showed that the carbon chain length of a subset of CHCs, the mbCHCs, can largely explain the differences in desiccation resistance across different species (*28*). In addition, phylogenetic comparative analysis showed that the evolution of longer mbCHCs in *Drosophila* species leads to evolution of higher desiccation resistance, with the desert *Drosophila* species, *Drosophila mojavensis* having the longest mbCHC lengths and the highest desiccation resistance across *Drosophila* species (*28*). Similarly, high proportions of very-long-chain mbCHCs are also observed in other desert insect species such as the desert tenebrionid beetle *Eleodes armata* (*29*), the desert locust *Schistocerca gregaria* (*30*), and the desert ant species *Cataglyphis niger* and *Pogonomyrmex barbatus* (*31*, *32*). These studies suggest that desert insect species may use long mbCHCs as a general mechanism to prevent water loss and increase desiccation resistance. However, it is not known whether the evolution of this trait is due to independent evolutionary changes in the same gene across these different desert-adapted species or changes in lineage-specific genes, leading to convergent evolution.

In this study, we investigated the genetic basis underlying desert adaptation in *D. mojavensis* (*10*, *33*). This species has adapted to several hot and dry deserts in southern California and Mexico (*34*), such as the Sonoran Desert where the relative humidity in the summer can be lower than 10% and the air temperature routinely exceeds 40°C (*35*). We showed that the synthesis of very long mbCHCs is necessary for the high desiccation resistance of *D. mojavensis* at these desert conditions. The ability to synthesize these very-long-chain mbCHCs is caused by coding differences in a fatty acyl–CoA elongase gene (*mElo*) that allows *D. mojavensis* to synthesize longer mbCHCs compared to *Drosophila melanogaster*, a well-studied cosmopolitan species. Phylogenetic analyses showed that *mElo* is a lineage-specific gene in *Drosophila*with no clear ortholog outside these species, suggesting that the evolution of high desiccation resistance in other desert insect species may be due to changes in the other genes.

## RESULTS

### The fatty acyl–CoA elongase *mElo* (*CG18609*) elongates mbCHCs in *D. melanogaster*

**Fig. 1.**
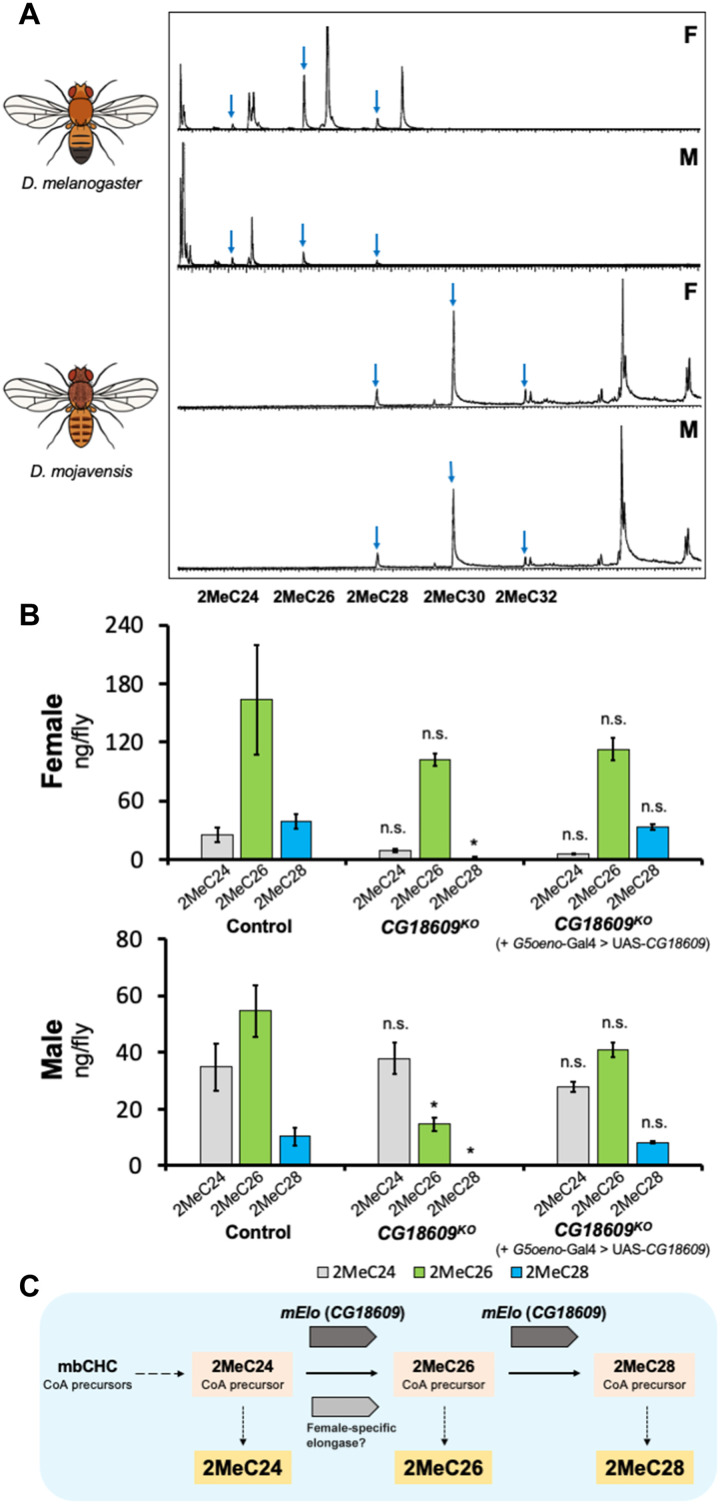
*mElo* (*CG18609*) is an mbCHC *elongase* in *D. melanogaster.* (**A**) Gas chromatography–mass spectrometry chromatograms showing female (F) and male (M) CHCs of *D. melanogaster* and *D. mojavensis*. The desert *Drosophila* species *D. mojavensis* produces longer mbCHCs than the cosmopolitan *D. melanogaster*. Blue arrows indicate mbCHCs in the chromatogram. (**B**) Levels of mbCHCs in *D. melanogaster CG18609* homozygous knockout (KO) and rescue strains with oenocyte-specific expression of *CG18609* (*G5*-Gal4 > UAS-*CG18609*) compared to the control strain *Cas9onIII*, which the knockout was derived from. In both sexes, the levels of 2MeC28 were significantly reduced (Student’s *t* test following with Benjamini-Hochberg correction at α = 0.05; female: *t*_7_ = 4.4, *P* = 0.03; male: *t*_7_ = 3.2, *P* = 0.02), while the level of 2MeC26 was only significantly reduced in males (*t*_7_ = 3.9, *P* = 0.02). No significant differences were observed in 2MeC24 in both sexes (female: *P* = 0.2; male: *P* = 0.9). The rescue strains were able to restore the production of mbCHCs in both sexes, leading to the mbCHC profiles that were not significantly different from the control strain (2MeC24, female: *P* = 0.08 and male: *P* = 0.7; 2MeC26, female: *P* = 0.1 and male: *P* = 0.4; 2MeC28, female: *P* = 0.5 and male: *P* = 0.4). n.s., not significant. *P < 0.05. (**C**) The role of *CG18609* (*mElo*) in the elongation of 2MeC24 to 2MeC26 and 2MeC28 in *D. melanogaster*, based on knockout and rescue data.

*Drosophila* species produce combinations of mbCHCs of different carbon backbone lengths ranging from 24 carbons (2MeC24) to 32 carbons (2MeC32) (*28*, *36*, *37*). *D. melanogaster* mainly produces 2MeC24, 2MeC26, and 2MeC28, while *D. mojavensis* produces longer mbCHCs, 2MeC28, 2MeC30, and 2MeC32 (Fig. 1A). The CHC synthesis pathway in insects takes place in specialized cells called oenocytes where acetyl-CoA is converted to CHCs by a series of enzymes including fatty acyl–CoA synthases, desaturases, reductases, elongases, and a P450 decarbonylase (*22*, *38*, *39*). In this pathway, three different types of enzymes could possibly account for differences in mbCHC production: fatty acyl–CoA synthases, reductases, and elongases. An mbCHC-specific fatty acyl–CoA synthase has been identified previously (*40*) but controls the early steps of mbCHC synthesis; thus, it would not be a candidate gene to account for the different mbCHC lengths between species. Fatty acyl–CoA reductases have been shown to affect CHC chain lengths but are not specific to a single type of CHC (*41*). As fatty acyl–CoA elongases are specific in determining the chain lengths of specific CHC types (*42*), we considered that a fatty acyl–CoA elongase specific to the elongation of mbCHCs could be a candidate gene underlying the differences in mbCHC production between *D. melanogaster* and *D. mojavensis*.

A previous genome wide association study in *D. melanogaster* showed that RNA interference of a specific fatty acyl–CoA elongase, *CG18609*, reduces the production of mbCHCs (*43*). To confirm the role of *CG18609* in the elongation of mbCHCs, we used CRISPR-Cas9 to knock out this gene in *D. melanogaster*. While homozygous *CG18609* knockout strains are viable and fertile, levels of 2MeC28 were significantly reduced in females, and both 2MeC26 and 2MeC28 were significantly reduced in males (Fig. 1B and table S1). Oenocyte-specific GAL4/UAS expression of a *D. melanogaster CG18609* transgene in the homozygous knockout strain was able to restore production of 2MeC26 and 2MeC28 (Fig. 1B). This suggests that *CG18609* is an elongase gene that is involved in the last elongation step in the synthesis pathway of the fatty acyl–CoA precursors for 2MeC26 and 2MeC28 in *D. melanogaster* (Fig. 1C). We named this gene *mElo* (*mbCHC elongase*).

### Transgenic overexpression of the *D. mojavensis mElo* (*Dmoj/mElo*) gene in *D. melanogaster* leads to longer mbCHC production and higher desiccation resistance

**Fig. 2.**
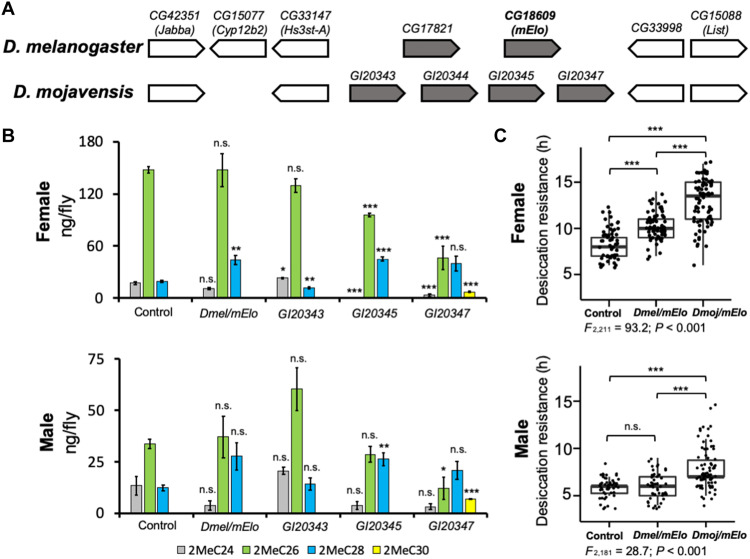
Oenocyte overexpression of *GI20347* in *D. melanogaster* leads to production of longer mbCHCs and confers higher desiccation resistance. (**A**) Microsynteny at the *mElo* locus is conserved between *D. melanogaster* and *D. mojavensis*. In *D. melanogaster*, two elongase genes, *CG17821* and *mElo*, are present at this locus. In *D. mojavensis*, four elongase genes (*GI20343*, *GI20344*, *GI20345*, and *GI20347*) are present. (**B**) Quantities of mbCHCs (in nanograms per fly) in *D. melanogaster* with each of the elongase genes (*mElo*, *GI20343*, *GI20345*, and *GI20347*) overexpressed in adult oenocytes using an oenocyte-specific driver. The quantity of each mbCHC in the overexpression strains was compared with the control to determine any significant differences using the Student’s *t* test following with Benjamini-Hochberg correction at α = 0.05. (**C**) Desiccation resistance of *D. melanogaster* strains with *mElo* and *GI20347* (*Dmoj/mElo*) overexpressed in the oenocytes. Desiccation resistance is measured in hours to mortality in a desiccating environment. Experiments were performed at 27°C for GAL4/UAS. Overexpression of *Dmoj/mElo* in *D. melanogaster* confers higher desiccation resistance in both males and females compared to control strains or strains with overexpression of *Dmel/mElo*. One-way analysis of variance (ANOVA) was used to determine the differences in desiccation resistance between the strains of *D. melanogaster*, following with post hoc comparisons using Tukey’s method. *P < 0.05; **P < 0.01; ***P < 0.001.

To investigate the molecular mechanisms underlying longer mbCHCs in *D. mojavensis*, we focused on the *mElo* gene of this species. At the *D. melanogaster mElo* locus, there are two elongase genes, *mElo* and another elongase gene, *CG17821*, while the *mElo* locus in *D. mojavensis* contains four predicted elongase genes, *GI20343*, *GI20344*, *GI20345*, and *GI20347* (Fig. 2A). Phylogenetic analyses suggest that *GI20347* is the ortholog of *mElo*, while *GI20343*, *GI20344*, and *GI20345* are likely to be orthologous with *CG17821* (fig. S1). We named *GI20347* as *Dmoj/mElo*. In situ hybridization with antisense probes of these genes showed that *mElo* is expressed in adult *D. melanogaster* oenocytes, while *GI20343*, *GI20345*, and *GI20347* (*Dmoj/mElo*) are expressed in adult *D. mojavensis* oenocytes. *CG17821* and *GI20344* are not expressed in *D. melanogaster* and *D. mojavensis* oenocytes, respectively (fig. S2).

To determine the function of these elongase genes in mbCHC production, we overexpressed *mElo*, *GI20343*, *GI20345*, and *GI20347* individually in adult *D. melanogaster* oenocytes using the GAL4/UAS system at 27°C. Overexpression of *GI20343* did not change mbCHC production in males but led to slightly reduced 2MeC28 and increased 2MeC24 in females (Fig. 2B and table S2). The overexpression of *mElo* in *D. melanogaster* led to an increase in 2MeC28 production in females (Fig. 2B and table S2), which is similar to what we have observed in *mElo* homozygous knockout flies (Fig. 1B), but we did not observe significant differences in males with *mElo* overexpression (Fig. 2B). Overexpression of *GI20343* and *GI20345* individually in the oenocytes altered proportions of 2MeC24, 2MeC26, and 2MeC28 produced but did not result in the production of any longer mbCHCs. However, when we overexpressed *GI20347* (*Dmoj/mElo*), we observed a shift to the production of longer CHCs, including the increased production of a longer mbCHC, 2MeC30, which is usually absent or present in trace amounts in *D. melanogaster* (Fig. 2B). As *GI20347* is the *D. mojavensis* ortholog of *D. melanogaster mElo*, we suggest that protein-coding differences in this elongase gene may underlie the differences in mbCHC production between these two *Drosophila* species.

These overexpression strains allowed us to test the hypothesis that the production of longer mbCHCs may confer higher desiccation resistance in *Drosophila* species, allowing species to survive under desert conditions. To test this, we performed desiccation assays on the strains with *Dmel/mElo* and *Dmoj/mElo* overexpression. Our experiments showed that transgenic *D. melanogaster* flies with *Dmoj/mElo* overexpression were significantly more desiccation resistant (means ± SE, females: 13.0 ± 0.3 hours, males: 7.8 ± 0.2 hours) compared to control flies (females: 8.4 ± 0.2 hours, males: 5.9 ± 0.1 hours) and flies with *Dmel/mElo* overexpression (females: 10.3 ± 0.2 hours, males: 6.0 ± 0.2 hours) (Fig. 2C). This result demonstrated that the production of longer mbCHCs can increase desiccation resistance, consistent with our previous findings using synthetic mbCHCs (*28*).

### *D. mojavensis mElo* (*Dmoj/mElo)* is necessary for high desiccation resistance at desert temperatures

**Fig. 3.**
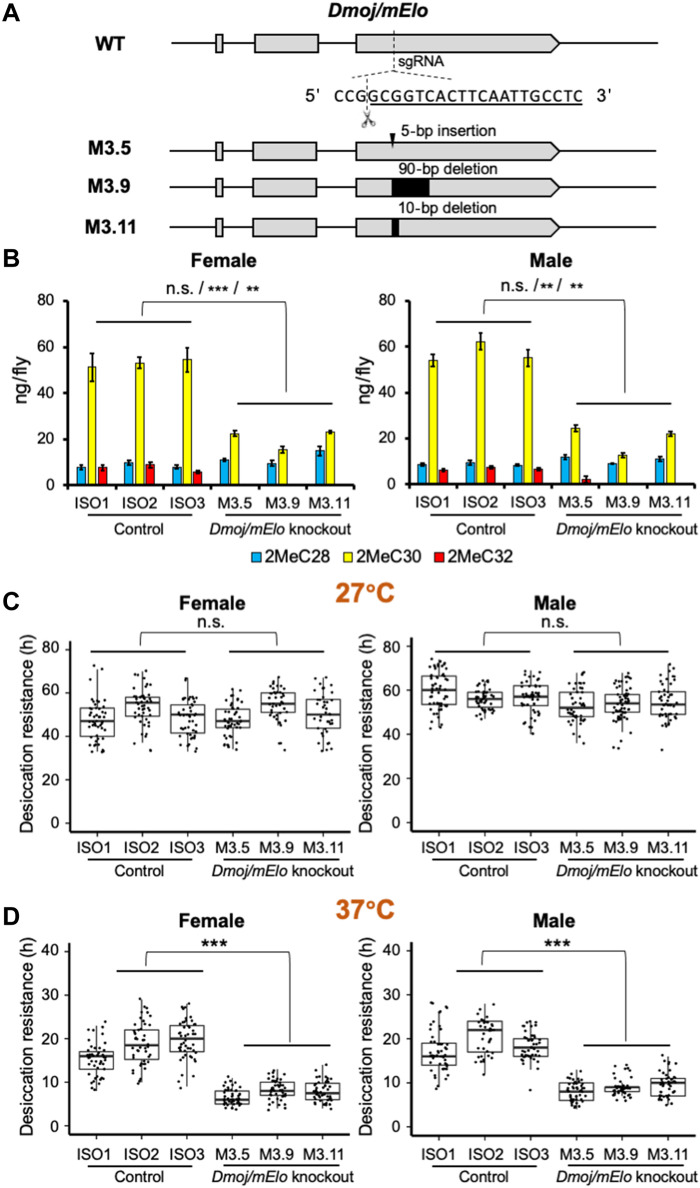
Knockout of the *mElo* ortholog *GI20347* (*Dmoj/mElo*) in *D. mojavensis* leads to a significant decrease in desiccation resistance at an ecologically relevant high temperature. (**A**) A CRISPR-Cas9 nonhomologous end-joining strategy resulted in three homozygous viable strains with *Dmoj/mElo* knockout, M3.5, M3.9, and M3.11 in *D. mojavensis*, which have a 5-bp insertion, a 90-bp deletion, and a 10-bp deletion, respectively. (**B**) In the three *Dmoj/mElo* (*GI20347*) knockout strains, 2MeC30 was significantly reduced [~50% of wild-type (WT) levels], and 2MeC32 is reduced to trace amounts (female: 2MeC30: *t*_4_ = −11.6, *P* < 0.001; 2MeC32: *t*_4_ = −8.5, *P* = 0.001; male: 2MeC30: *t*_4_ = −8.5, *P* = 0.001; 2MeC32: *t*_4_ = −7.3, *P* = 0.002). (**C**) There are no significant differences in desiccation resistance between the three *Dmoj/mElo* knockout strains and the three isofemale control strains at 27°C (female: *P* = 0.7; male: *P* = 0.06). (**D**) At 37°C, the three *Dmoj/mElo* knockout strains have a significant reduction in desiccation resistance compared to the three isofemale control strains (female: *t*_4_ = 7.4, *P =* 0.002; male: *t*_4_ = 9.5, *P* < 0.001). For both CHC quantities and desiccation resistance, linear mixed-effects models were applied to compare the two groups of flies using *lmer* function in R (version 4.1). The three isofemale wild-type and independent knockout strains were included as random effects. The difference between the wild-type and knockout flies was determined by paired contrast at α = 0.05. **P < 0.01; ***P < 0.001.

While our experiments showed that transgenic overexpression of *Dmoj/mElo* in *D. melanogaster* produces longer mbCHCs such as 2MeC30 and confers higher desiccation resistance, we did not recapitulate the production of 2MeC32 and the very high desiccation resistance in the desert dwelling *D. mojavensis* (*28*). To investigate the role of *Dmoj/mElo* in mbCHC synthesis and desiccation resistance in *D. mojavensis*, we used CRISPR-Cas9 to knock out *Dmoj/mElo* in *D. mojavensis*. Three independent *Dmoj/mElo* knockout strains, M3.5, M3.9, and M3.11, carrying a 5–base pair (bp) insertion, 90-bp deletion, and 10-bp deletion in the exon 3 of *Dmoj/mElo*, respectively, were obtained (Fig. 3A and fig. S3). All three mutant strains are homozygous viable. We also established three independent isofemale strains, ISO1, ISO2, and ISO3, from the parental population as controls.

In all three *Dmoj/mElo* knockout strains, 2MeC32, the longest mbCHC in *D. mojavensis*, was reduced to trace amounts, and 2MeC30 was significantly reduced compared to the control strains (Fig. 3B, fig. S4, and table S3), suggesting that *Dmoj/mElo* is responsible for elongating 2MeC28 to 2MeC30 and 2MeC32 in *D. mojavensis*. We further examined how these changes in mbCHC lengths could affect desiccation resistance of *D. mojavensis* by subjecting all knockout and control strains to the desiccation assay at 27°C. However, we did not observe any significant difference in desiccation resistance between the knockout strains and the controls at this temperature (Fig. 3C). As the capability of CHCs in preventing water loss is associated with their melting temperatures (*44*, *45*) and the air temperature of the microhabitat of *D. mojavensis* (e.g., outside cactus necrosis in the Sonoran Desert) is higher than 35°C (*35*), we considered the hypothesis that at these higher temperatures, longer mbCHCs such as 2MeC32 may make a difference in desiccation resistance. Therefore, we tested whether the reduced 2MeC30 and 2MeC32 in *Dmoj/mElo* knockout *D. mojavensis* could affect its desiccation resistance at 37°C, a temperature that is ecologically relevant to *D. mojavensis*.

Desiccation experiments at 37°C showed that across the board, time to mortality is faster than experiments performed at 27°C. At this temperature, the three *Dmoj/mElo* knockout strains are significantly less desiccation resistant (females: 7.7 ± 0.2 hours, males: 8.9 ± 0.2 hours) compared to the control strains (females: 18.0 ± 0.4 hours, males: 18.6 ± 0.4 hours) (Fig. 3D), suggesting that the production of longer mbCHCs such as 2MeC30 and 2MeC32 is crucial in desiccation resistance under hot and dry conditions. To exclude the possibility that the higher mortality of the *Dmoj/mElo* knockout flies compared to the control flies was due to heat stress rather than increased water loss at 37°C, we tested the survival of adults of both knockout and control strains at 37°C under a standard laboratory environment (the flies were given fresh food every 1 to 2 days because flies obtain water from the food). Survival at 37°C between the control strains and the *Dmoj/mElo* knockout strains was not significantly different under these conditions (fig. S5), suggesting that the increased mortality observed during the desiccation experiment at 37°C was due to water loss rather than the higher temperature. Together, our results demonstrated that in *D. mojavensis*, *Dmoj/mElo* underlies the production of long mbCHCs such as 2MeC30 and 2MeC32 and contributes to the high desiccation resistance of this species in its hot and arid desert environment.

### The *mElo* gene is a *Drosophila*-specific mbCHC elongase

**Fig. 4.**
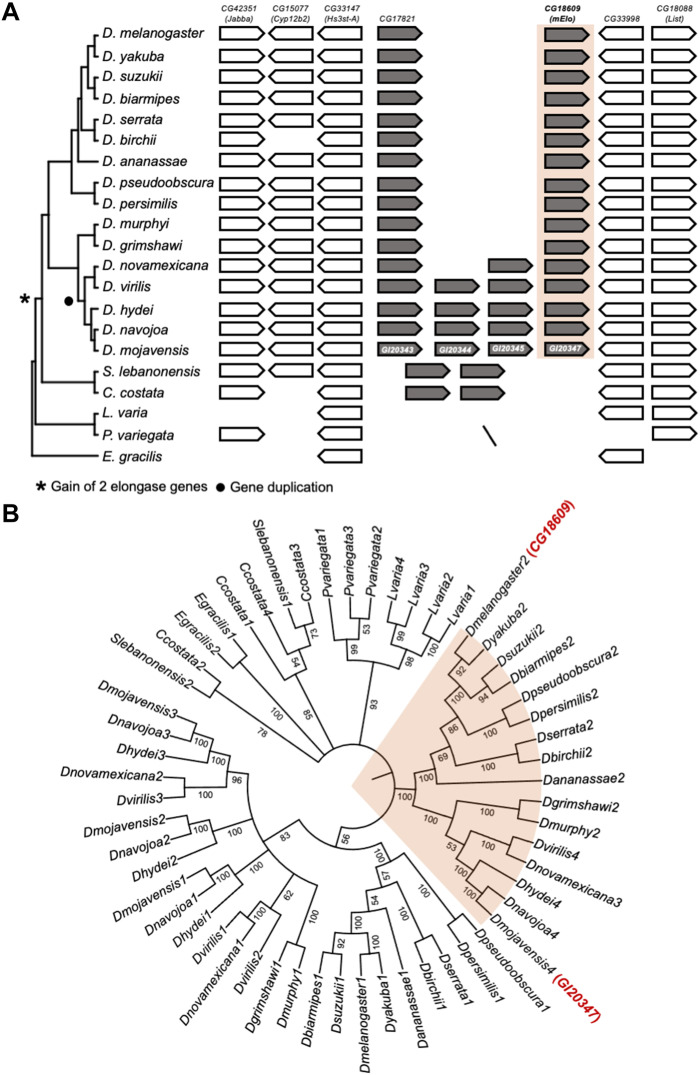
The origins of *mElo* in *Drosophila*. (**A**) The *mElo* loci in 16 *Drosophila* species and species from five closely related genera. The *mElo* loci were identified on the basis of the conserved genes in the *D. melanogaster mElo* locus (*Jabba*, *Cyp12b2*, *Hs3st-A*, *CG33998*, and *List*) that are used as anchor genes in our analysis. All *Drosophila* species contains at least two elongase genes at this locus. There was an expansion of elongase genes in the virilis and repleta clades where species have three to four elongase genes at this locus (denoted by a solid circle). Two elongase genes are present in *S. lebanonensis* and *C. costata*, but none in *L. varia*, *P. variegata*, and *E. gracilis.* This suggests that elongase genes at this locus first originated in the common ancestor of the *Drosophila*, *Scaptodrosophila*, and *Chymomyza* genera (denoted by an asterisk). The backslash line indicates that anchor genes can be located in the genome but at different locations. (**B**) Phylogenetic relationship of elongase genes in *mElo* loci of Drosophilinae species and the elongases from *S. lebanonensis*, *C. costata*, *L. varia*, *P. variegata*, and *E. gracilis* that share the highest similarities to *mElo*. The phylogenetic tree was inferred by the maximum likelihood method using amino acid sequences with 1000 bootstrap tests. The numbers next to nodes represent bootstrap values.

As mbCHCs are almost ubiquitous in most insect species, we sought to investigate whether the role of *Dmoj/mElo* in determining mbCHC length and desiccation resistance is conserved across Insecta. Using the conserved microsynteny (*Jabba*, *Cyp12b2*, *Hs3st-A*, *CG33998*, and *List*) around *CG17821* and *mElo* between *D. melanogaster* and *D. mojavensis*, we investigated this locus in 16 *Drosophila* species and 5 species from closely related genera (*Scaptodrosophila*, *Chymomyza*, *Leucophenga*, *Phortica*, and *Ephydra*) (Fig. 4A) (*46*–*48*). We found that in the *Drosophila* species examined, this microsynteny is conserved and the elongase gene copy number ranges from two to four across these *Drosophila* species. This microsynteny is also conserved in *Scaptodrosophila lebanonensis*, *Chymomyza costata*, and *Leucophenga varia* and partially conserved in *Phortica variegata*, *Ephydra gracilis*, and *Musca domestica.* There are two elongase genes at this locus in *S. lebanonensis* and *C. costata* but none in *L. varia*, *P. variegata*, and *E. gracilis.* This suggests that elongase genes at this locus first originate in the common ancestor of the *Drosophila*, *Scaptodrosophila*, and *Chymomyza* genera (i.e., the Drosophilinae subfamily) (Fig. 4A).

To determine the relationship of these elongase genes, we performed a phylogenetic analysis of all the elongase genes at the *mElo* loci from 16 *Drosophila* species, *S. lebanonensis*, and *C. costata*. We also included elongase genes in *L. varia*, *P. variegata*, and *E. gracilis* that have the highest sequence homology to the elongases at the *Drosophila mElo* locus. Phylogenetic analysis of all elongase genes in *mElo* loci showed that each *Drosophila* species only has a single *mElo* ortholog (Fig. 4B). In addition, the elongase genes in *Scaptodrosophila* and *Chymomyza* did not cluster with those in *Drosophila* (Fig. 4B), suggesting that the presence of multiple elongase genes in the two lineages is likely due to lineage-specific gene duplication events. This result suggests that the *mElo* gene at the *Drosophila mElo* locus originated in the genus *Drosophila*. However, this does not exclude the possibility that the *mElo* gene is present in other insect species but located in another genomic location, as mbCHCs are prevalent across insect species. To determine whether any *mElo* ortholog is present in other insect species, we compared elongase genes in *Aedes aegypti*, a Dipteran mosquito species, and several non-Diptera species, *Apis mellifera*, *Bombyx mori*, and *Tribolium castaneum.* From our phylogenetic analysis, we observed that while other elongase genes such as *bond*, *sit*, and *CG31523* have 1:1 ortholog in these insect species, there is no clear *mElo* orthologous gene identified (fig. S6). This suggests that the *mElo* gene is a *Drosophila*-specific mbCHC elongase and other elongase genes may elongate mbCHCs in other insect species.

## DISCUSSION

A few of the many species on Earth have evolved adaptive traits to live in extreme environments with harsh abiotic conditions. However, few studies have determined the underlying genetic basis for these traits. In this study, we show that the desert *Drosophila* species, *D. mojavensis*, has evolved coding changes in a fatty acyl–CoA elongase gene, *mElo*, which led to the production of very long mbCHCs and high desiccation resistance in this species. While the knockout of this gene in *D. mojavensis* has no significant effects on desiccation resistance at a lower temperature (27°C), it significantly reduces desiccation resistance at a higher temperature (37°C), which is within the average high temperature range in the Sonoran Desert during summer (fig. S7). This suggests that these very long mbCHCs are able to reduce water loss at hot-arid desert conditions, i.e., high temperature and low humidity, and are crucial for the survival of *D. mojavensis* in this habitat. The transgenic overexpression of the *D. mojavensis mElo* gene in the cosmopolitan *D. melanogaster* led to the production of longer mbCHCs and higher desiccation resistance compared to the transgenic overexpression of the *D. melanogaster mElo* gene, suggesting evolved coding differences in this gene between the two species. Last, phylogenetic analyses of this locus suggest that the *mElo* gene evolved recently and an orthologous copy of this gene is not found outside Diptera.

### The critical roles of very long mbCHCs for the survival of *D. mojavensis* in hot and arid deserts

Why are there differences in desiccation resistance at 37°C but not at 27°C between *Dmoj/mElo* knockout strains and the control strains? The CHC layer is a solid-liquid mixture, and its ability to prevent water loss depends on the physical properties such as the phase and melting behavior, which affects its ability to prevent water molecules from diffusing through (*49*). At a specific “phase transition” temperature, the CHC layer transits to a more liquid status and water loss through the insect cuticle increases rapidly (*24*, *45*). This transition temperature differs among species and is determined by the CHC composition of each species (*24*, *49*).

**Fig. 5.**
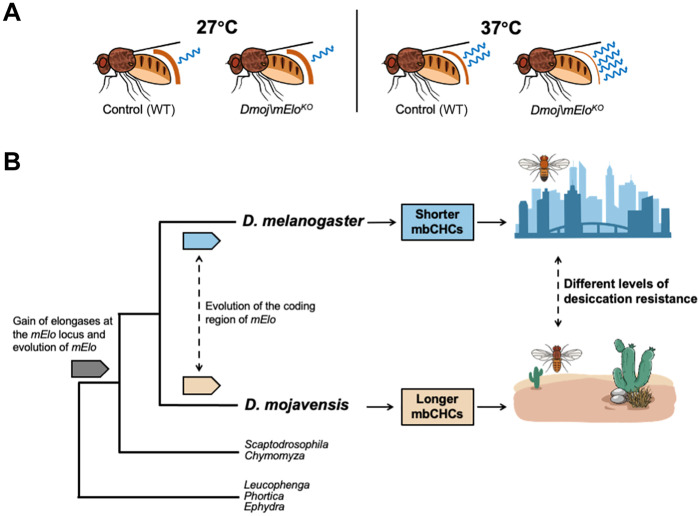
Evolution of a fatty acyl–CoA elongase gene, *mElo*, underlies higher desiccation resistance and desert adaptation in *D. mojavensis*. (**A**) A schematic showing that the loss of 2MeC32 and a notable amount of 2MeC30 at 27°C do not affect the *Dmoj/mElo* strain of *D. mojavensis* compared to the control strain as water loss is similar between these strains. However, at the higher temperature of 37°C, we hypothesized the *Dmoj/mElo* strain loses water more rapidly compared to the control strain due to the melting temperatures of the CHC layer being altered by the loss of these longer mbCHCs. (**B**) A model showing how coding changes in *mElo* led to shorter mbCHCs in the cosmopolitan species *D. melanogaster* and longer mbCHCs in the desert species, *D. mojavensis*, allowing it to survive in the hot and dry desert.

We suggest that the loss of 2MeC32 and the decrease in 2MeC30 in the *Dmoj/mElo* knockout strains altered the transition temperature of the CHC layer on *D. mojavensis*. At 27°C, this does not affect the *Dmoj/mElo* knockout strains; therefore, they do not differ in desiccation resistance from the control strains. However, at 37°C, *Dmoj/mElo* knockout strains begin to lose water more rapidly than the control strains, resulting in a decrease in desiccation resistance compared to the control strains (Fig. 5B). As hot and arid deserts have long days of high temperatures during the summer, we suggest that the very long mbCHCs in *D. mojavensis* are crucial for survival as they allow *D. mojavensis* to survive the hot and dehydrating conditions during the long day before the dip in temperatures during the night.

### Evolution at the *mElo* locus in *Drosophila*

The oenocyte driven overexpression of *Dmel/mElo* and *Dmoj/mElo* in the *D. melanogaster* produces mbCHCs of different chain lengths (Fig. 2), suggesting that there are differences in protein-coding sequences of this gene between the two *Drosophila* species and that these differences contribute to the different mbCHCs produced by these two species. Our previous study using ancestral trait reconstruction showed that the last common ancestor of *D. melanogaster* and *D. mojavensis* has an mbCHC phenotype that is intermediate between the mbCHCs phenotypes of the two species (*28*). As *mElo* controls the length of the longest mbCHCs produced in each of these two species, this suggests that evolutionary changes in this gene may have occurred in both species from the common ancestor, i.e., evolution in this gene led to *D. melanogaster* to produce shorter mbCHCs and *D. mojavensis* to produce longer mbCHCs as both species adapt to their environments (Fig. 5B).

The CRISPR-Cas9 knockout of *mElo* in both species also produced different mbCHC phenotypes. In *D. melanogaster*, knockout of *mElo* produced an mbCHC phenotype that is largely 2MeC24 in males and 2MeC26 in females with decreases in 2MeC28 in both sexes compared to wild-type flies. In *mElo* knockout *D. mojavensis*, while 2MeC30 and 2MeC32 are reduced with the latter reduced to trace amounts compared to wild-type flies, the major CHC in these *mElo* knockout *D. mojavensis* is still 2MeC30. This suggests that there are other elongase genes contributing to the mbCHC phenotype in *D. mojavensis*. A candidate gene for this would be *GI20345*, another elongase gene in the *mElo* locus in *D. mojavensis* that is expressed in *D. mojavensis* oenocytes and is able to elongate mbCHCs in *D. melanogaster* (Fig. 2). This may suggest a complicated evolutionary scenario in the evolution of mbCHC biosynthesis controlled by the *mElo* locus in *Drosophila* (fig. S8).

### Lineage-specific genetic basis for the evolution of desiccation resistance

Variations in CHC composition contribute to differences in desiccation resistance across many insect species (*28*, *50*–*52*). While mbCHCs are found in almost all insect species, our phylogenetic analyses showed that the *mElo* gene is a *Drosophila*-specific mbCHC elongase, indicating that the control of mbCHC chain length in other insect species is likely through a different elongase gene. This suggests that the contribution to the evolution of higher desiccation resistance and desert adaptation by the *mElo* locus is likely to be a mechanism specific to the *Drosophila* lineage. If changes in CHC composition can contribute to desiccation resistance in insects, then what are the likely genetic mechanisms that underlie the evolution of desiccation resistance outside the *Drosophila* lineage? The CHC biosynthesis pathway is largely conserved in insects and is made up of several fatty acyl–CoA synthesis gene families such as fatty acyl–CoA synthases, desaturases, reductases, and elongases (*22*). These gene families evolved rapidly and contribute to the diversification of CHCs across insects (*53*–*56*). Gains and losses of these genes as well as changes in their oenocyte expression are likely to contribute to CHC changes and the evolution of desiccation resistance in different insect species. The rapid “birth-and-death” of these genes also suggests that many of the genetic mechanisms leading to CHC changes and the evolution of desiccation resistance across different insect species are likely to be lineage specific.

In summary, we showed that evolutionary change in a fatty acyl–CoA elongase contributes to the adaptation of *D. mojavensis* to the hot and arid Sonoran Desert by reducing water loss at a high temperature. While there are similar physiological mechanisms in many insect species in evolving CHC layers with higher melting temperatures to prevent water loss under hot and arid conditions, the genetic mechanisms underlying these changes may be species specific or lineage specific. This may have implications for the prediction of species changes as climate change continues to occur in the near future.

## MATERIALS AND METHODS

### *Drosophila* strains

The *y w; attP40* strain was used for in situ hybridization and transgenesis in *D. melanogaster*. The *D. mojavensis wrigleyi* strain (15081-1352.29) used was obtained from the National Drosophila Species Stock Center. The *oeno*GAL4 strain [*PromE(800) line 2 M*] was a gift from J. Levine (*57*). The balancer strain *w^1118^; CyO/Sco; MKRS/TM6B, Tb^1^* (#3703) and y^1^ w^*^ P{y^+t7.7^ = nos-phiC31\int.NLS}X; CyO/Sco (#34770) were obtained from the Bloomington *Drosophila* Stock Center. All flies were maintained at room temperature on standard *Drosophila* food (Bloomington formulation, Genesee Scientific). *D. melanogaster* GAL4/UAS experiments were performed at 27°C.

### In situ hybridization and imaging

In situ hybridization was performed on oenocytes of 5-day-old adults using methods as described previously (*58*, *59*). Primers that were used for synthesizing probes were listed in table S4. All in situ hybridization images were captured using the Nikon SMZ18 dissecting stereo microscope system.

### Generation of *mElo* knockout by CRISPR-Cas9 genome engineering in *D. melanogaster*

CRISPR-Cas9–mediated homology-directed repair was used to generate a knockout of *Dmel/mElo*. The program, flyCRISPR Optimal Target Finder, was used to identify optimal CRISPR target sites (*60*). Target-specific sequences for *Dmel/mElo* were synthesized as oligonucleotides, phosphorylated, annealed, and ligated into the BbsI sites of *pU6–*BbsI*–chiRNA* (Addgene plasmid #45946) (*61*) (5′: *Dmel/mElo*-gRNA1–BbsI–F and *Dmel/mElo*-gRNA1–BbsI–R; 3′: *Dmel/mElo*-gRNA2–BbsI–F and *Dmel/mElo*-gRNA2–BbsI–R). To construct the replacement donor, approximately 1-kb homology arms flanking the cut sites were amplified by polymerase chain reaction (PCR) using primers *Dmel/mElo*-RightHomo–AscI–F and *Dmel/mElo*-RightHomo–XhoI–R for the 5′ homology arm and primers *Dmel/mElo*-LeftHomo–EcoRI–F and *Dmel/mElo*-LeftHomo–NotI–R for the 3′ homology. The replacement donors were cloned sequentially into the corresponding cut sites of the double-stranded DNA donor vector *pHD-DsRed-attP* (Addgene plasmid #51019). The primers used for generating gRNA and replacement donor constructs are listed in table S4. The two guide RNA (gRNA) constructs and the replacement donor construct were coinjected into the w^1118^; PBac{y^+mDint2^ GFP^E.3xP3^ = vas-Cas9}VK00027 strain (denoted as *Cas9onIII* strain; BDSC #51324), which carries a *vasa-Cas9* transgene on chromosome 3. The DsRed fluorescence in the eyes was used to screen positive progeny, which was then crossed to *w^1118^* to remove the *vasa-Cas9* transgene before being back-crossed for five generations and then made homozygous using the double balancer strain *w^1118^; CyO/Sco; MKRS/TM6B, Tb^1^*. The replacement of *Dmel/mElo* with *attP*/*DsRed* by homology-directed repair was confirmed by PCR using the primers *DmelCG18609*–EcoRI–F and *DmelCG18609*–XbaI–R and the presence of DsRed (fig. S9A). The resulting strain is designated as *w^1118^; CG18609^KO-DsRed-attP^* (*mEloKO*). A transgene carrying a *PhiC31* integrase driven by a *nanos* enhancer was integrated into this strain by crossing it to *y^1^*,w*,*P{y^+t7.7^ = nos-phiC31\int.NLS}X; Sco/CyO* (fig. S9B). The resulting strain is *w^1118^*, *P{y^+t7.7^ = nos-phiC31\int.NLS}X; CG18609^KO-DsRed-attP^* and named as the *mEloKOint* strain.

### Generation of plasmid constructs

Primers used for generating all constructs are listed in table S4. UAS overexpression constructs were cloned in *PhiC-31* site–specific transformation vector, *pWalium10-MOE* (*62*). The genomic DNA of *Dmel/CG17821*, *Dmel/CG18609* (*Dmel/mElo*), *Dmoj/GI20343*, *Dmoj/GI20344*, *Dmoj/GI20345*, and *Dmoj/GI20347* (*Dmoj/mElo*) were amplified by PCR from genomic DNA of corresponding species and then cloned into *pWalium10-MOE* vector using the NdeI, EcoRI, or XbaI sites. The *G5-GAL4* construct was made by cloning the 5′ regulatory region of *Dmoj/GI20345* into the green fluorescent protein (GFP) reporter vector *pS3aG* via the AscI and SbfI sites (*63*). The GFP sequence was then cut out from this construct using SpeI and SbfI and replaced with a GAL4 sequence *pBPGUw* (Addgene plasmid #17575) vector using SpeI and SbfI.

### *Drosophila *transgenesis and overexpression experiments

Transgenesis in *D. melanogaster* (*y w; attP40* and *mEloKOint* strains) was performed using the *PhiC31* integrase system following standard *Drosophila* transgenesis protocols. To generate UAS overexpression strains, the overexpression constructs of elongase genes in *pWalium10-MOE* were individually injected into the *y w; attP40* strain. The *G5-*GAL4 construct and the overexpression construct of *Dmel/mElo* on *pWalium10-MOE* were individually injected into the *mEloKOint* strain for the rescue of *mElo* expression in *mElo* knockout *D. melanogaster*. All overexpression experiments were performed at 27°C by reciprocally crossing *oeno*GAL4 strain (*oeno*GAL4 or *G5-*GAL4) and the corresponding UAS overexpression strain.

### Generation of *mElo* knockout by CRISPR-Cas9 genome engineering in *D. mojavensis*

To generate *Dmoj/mElo* mutant alleles in *D. mojavensis*, we used a nonhomologous end joining mediated strategy by injecting the mixture of Cas9 protein (PNA Bio, #CP01) and single-guide RNAs (sgRNAs) into the embryos of this species. We coinjected two sgRNAs targeting *Dmoj/white* (*Dmoj/white*_sgRNAa and *Dmoj/white*_sgRNAb) (*37*). *Dmoj/mElo* specific sgRNAs (*Dmoj*/*mElo*-sgRNAa and *Dmoj/mElo*-sgRNAb) were designed using the online tool CRISPR Design (*61*), and two sgRNAs were selected. All sgRNAs were generated with in vitro transcription using T7 Megascript Kit (Ambion) and purification using a MegaClear Kit (Ambion) (*64*). Primers used for the synthesis of all sgRNAs were listed in table S4. The final injection mixture is composed of Cas9 protein (300 ng/μl) and four sgRNAs, each 75 ng/μl. To screen for the offspring of *D. mojavensis* carrying *Dmoj/mElo* mutant alleles, we used the T7E1 assay (NEB, #E3321) to determine potential mutations for every single fly following the protocol in (*65*). To eliminate potential off-targets from the gene knockout, all strains carrying mutations in *Dmoj/mElo* were backcrossed with the parental *D. mojavensis* strain for at least five generations before being made homozygous.

### CHC extraction and analyses

CHC extraction, gas chromatography–mass spectrometry analysis, CHC identification, and quantification were performed as described previously (*28*). The gas chromatography thermal program was set as follows: start from 100°C, 5°C/min to 200°C, and 3°C/min to 325°C. For each sex in each reciprocal cross, two to four extractions were conducted as replicates, and the results were pooled for further statistical analyses, so four to seven replicates from each cross were conducted.

### Desiccation assay

Desiccation assays were performed as described previously (*28*). Silica gel (S7500-1KG) was ordered from Sigma-Aldrich. For each genotype, six replicates were conducted, each three from each reciprocal cross.

### Bioinformatics

The sequences of all elongase genes used in this study were retrieved from the National Center for Biotechnology Information (www.ncbi.nlm.nih.gov) database, VectorBase (*66*), and SilkDB (*67*) via TBLASTN using *CG17821* and *CG18609* as queries (dataset S1). The DNA or amino acid sequences were aligned with MUSCLE and manually adjusted for the phylogenetic reconstruction using the maximum likelihood method in MEGA (version 11) (*68*). The Generalized Time Reversible (GTR) model with a gamma distribution was applied to reconstruct phylogeny using protein coding sequences of elongase genes, while the LG substitution matrix and a gamma distribution with invariant sites (G + I) was applied using their amino acid sequences. All phylogenetic reconstruction analyses used 1000 bootstrap replicates to test the reliability of inferred trees. The phylogenetic relationship of *Drosophila* and related species used in this study was adapted from (*59*, *69*).

### Statistics

Differences in CHCs between each transgenic *D. melanogaster* line and the control were compared using Student’s *t* test. To account for multiple testing from different CHCs, the *P* values were adjusted using Benjamini-Hochberg correction at α = 0.05. Differences in desiccation resistance between *D. melanogaster* overexpression lines were determined using one-way analysis of variance (ANOVA) with post hoc comparisons using Tukey’s method at α = 0.05. For CHCs and desiccation resistance between the wild-type *D. mojavensis* and *mElo* knockout *D. mojavensis* lines, a linear mixed-effects model was applied, and the specific lines within each group were incorporated as random effects. All analyses were performed in R (version 4.1.3).
