# Supplementary material for: Evolution of a fatty acyl–CoA elongase underlies desert adaptation in Drosophila
Source: Sci Adv. 2023 Aug 30;9(35):eadg0328. doi: 10.1126/sciadv.adg0328 (PMC10468142; doi:10.1126/sciadv.adg0328)
Supplement: Supplementary file 1 — Figs. S1 to S9 Tables S1 to S4 Legend for dataset S1 [file sciadv.adg0328_sm.pdf]

Science Advances
AAAS

Supplementary Materials for

Evolution of a fatty acyl–CoA elongase underlies desert adaptation in *Drosophila*

Zinan Wang *et al.*

Corresponding author: Henry Chung, hwchung@msu.edu

Sci. Adv. **9**, eadg0328 (2023)
DOI: 10.1126/sciadv.adg0328

The PDF file includes:

Figs. S1 to S9
Tables S1 to S4
Legend for dataset S1

Other Supplementary Material for this manuscript includes the following:

Dataset S1

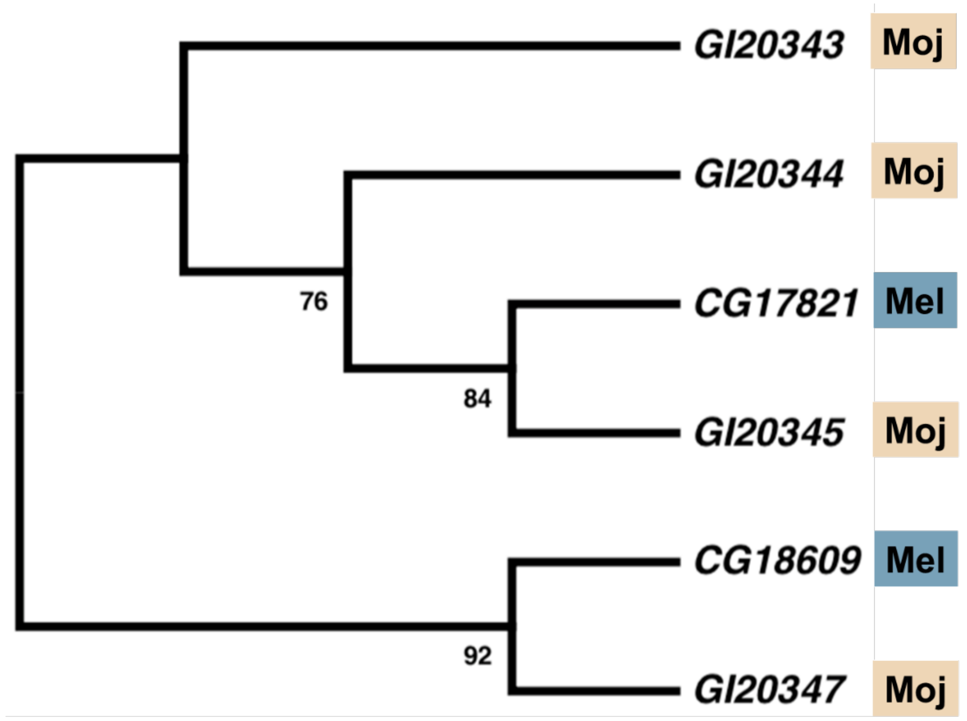

Figure S1. Phylogeny of elongases in the *mElo* loci of *D. melanogaster* and *D. mojavensis.* The coding sequences of these genes were used to generate the phylogeny using the Maximum Likelihood method with the GTR model and 1000 bootstraps. The phylogenetic analysis showed that the *D. mojavensis* orthologue of *mElo* is *GI20347*.

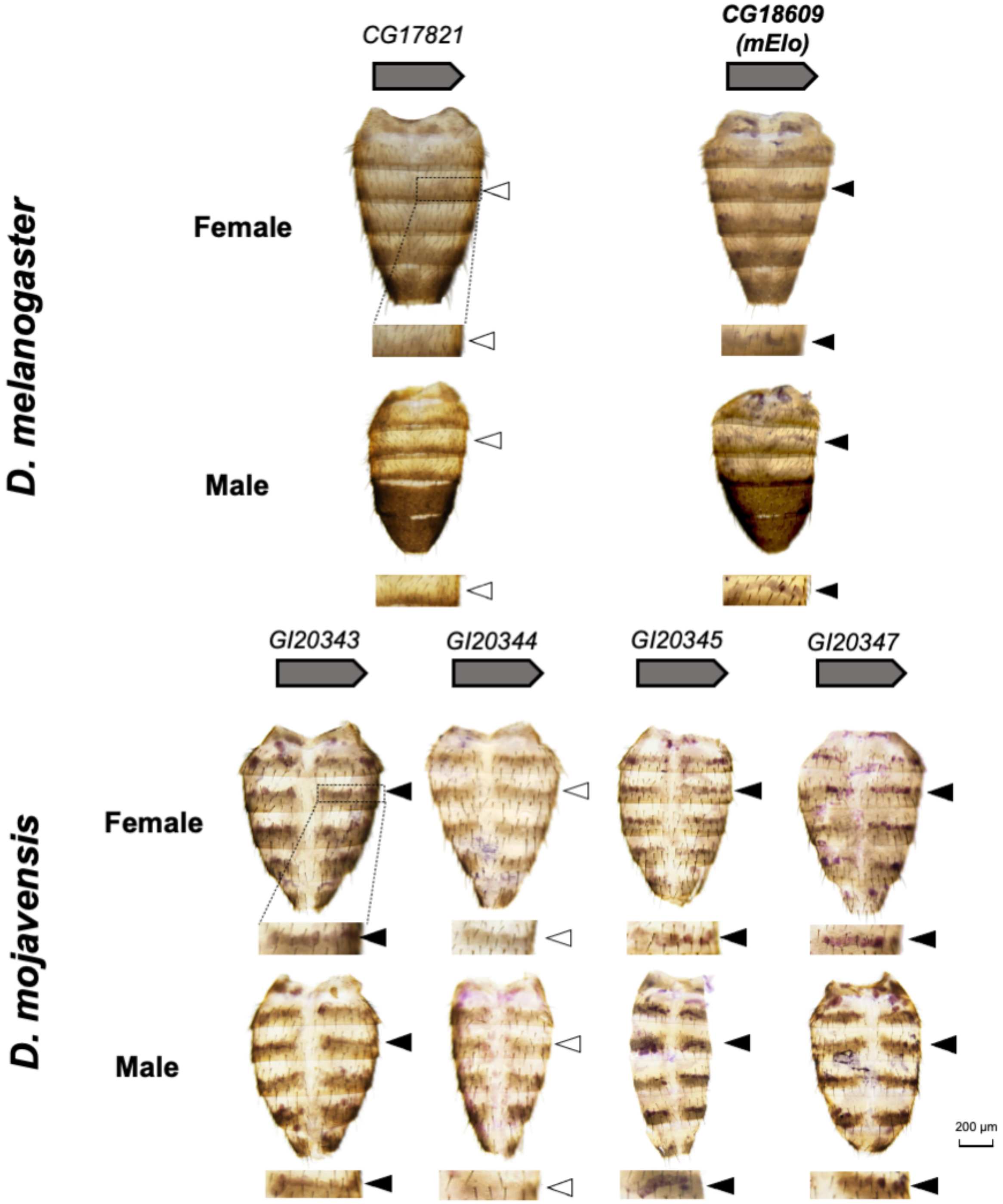

Figure S2. RNA *in situ* hybridization of fatty acyl-CoA elongase genes in the *D. melanogaster* and the *D. mojavensis mElo* loci on adults. In *D. melanogaster*, *CG18609* RNA transcript was detected in the adult oenocytes. In *D. mojavensis*, *GI20343*, *GI20345*, and *GI20347* RNA transcripts were detected in the adult oenocytes. The expressions of all four genes are sexually monomorphic. Arrowheads point to oenocytes. Filled arrowheads indicate visible expression detected and open arrowheads indicate no visible expression.

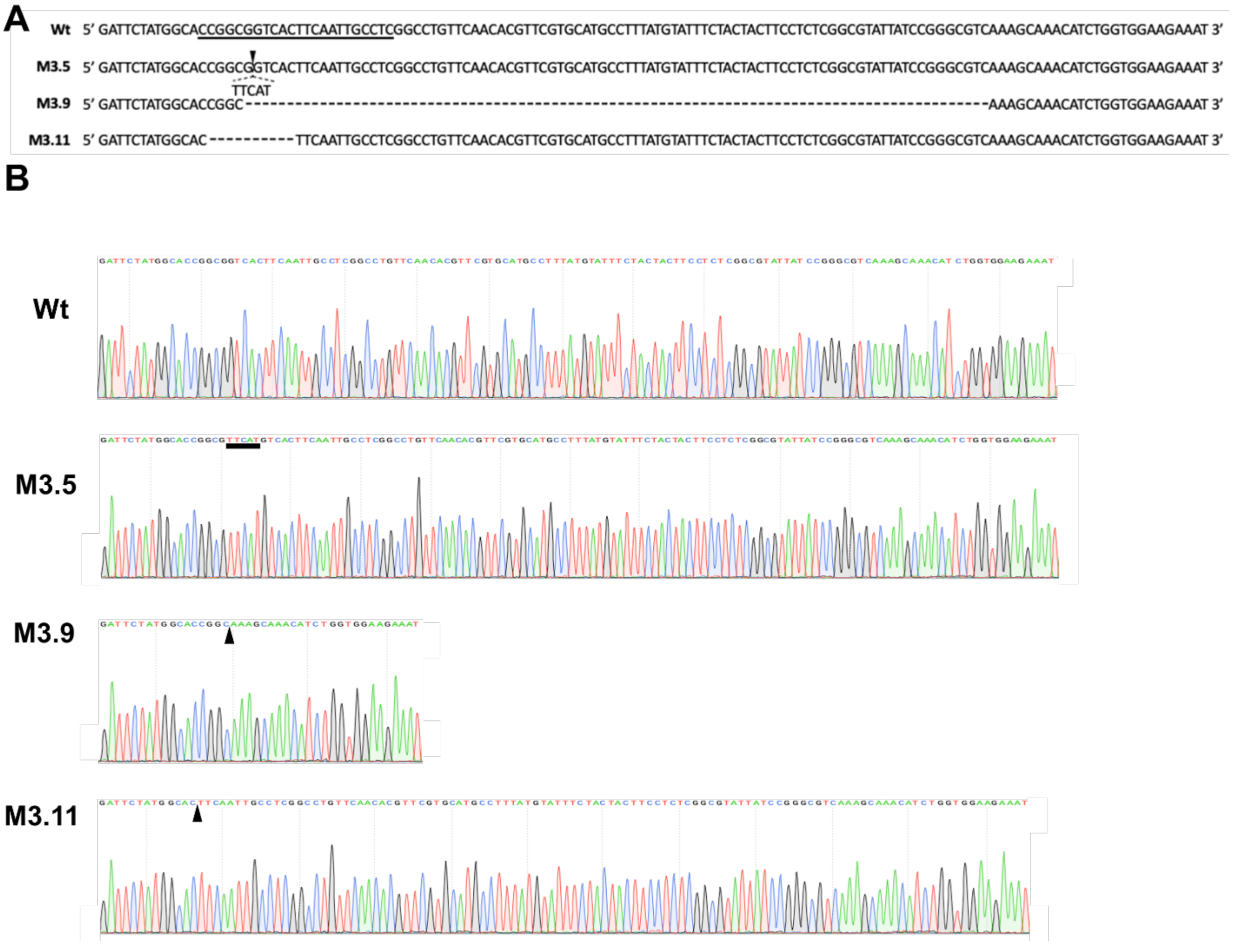

Figure S3. Edited sequences in M3.5, M3.9, and M3.11 strains. CRISPR/Cas9 and non-homologous end-joining was used to generate knockout strains of *GI20347*. Three independent knockout strains, namely M3.5, M3.9, and M3.11, were generated. They carry a 5-bp insertion, 90-bp deletion, and 10-bp deletion in the exon 3 of *Dmoj/mElo*, respectively.

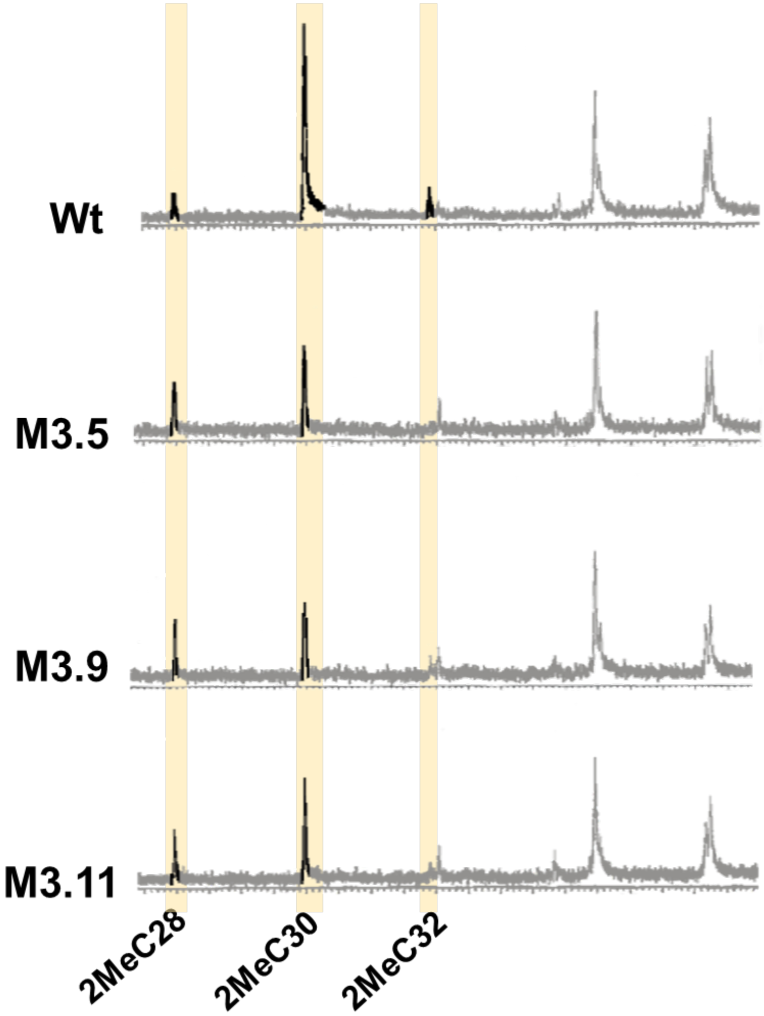

Figure S4. GC-MS chromatograms of mbCHCs in three homozygous *Dmoj/mElo* knockout strains of *D. mojavensis,* M3.5, M3.9, and M3.11*.* In all three knockout strains, levels of 2MeC30 and 2MeC32 were reduced and levels of 2MeC28 were increased compared to the wild-type control.

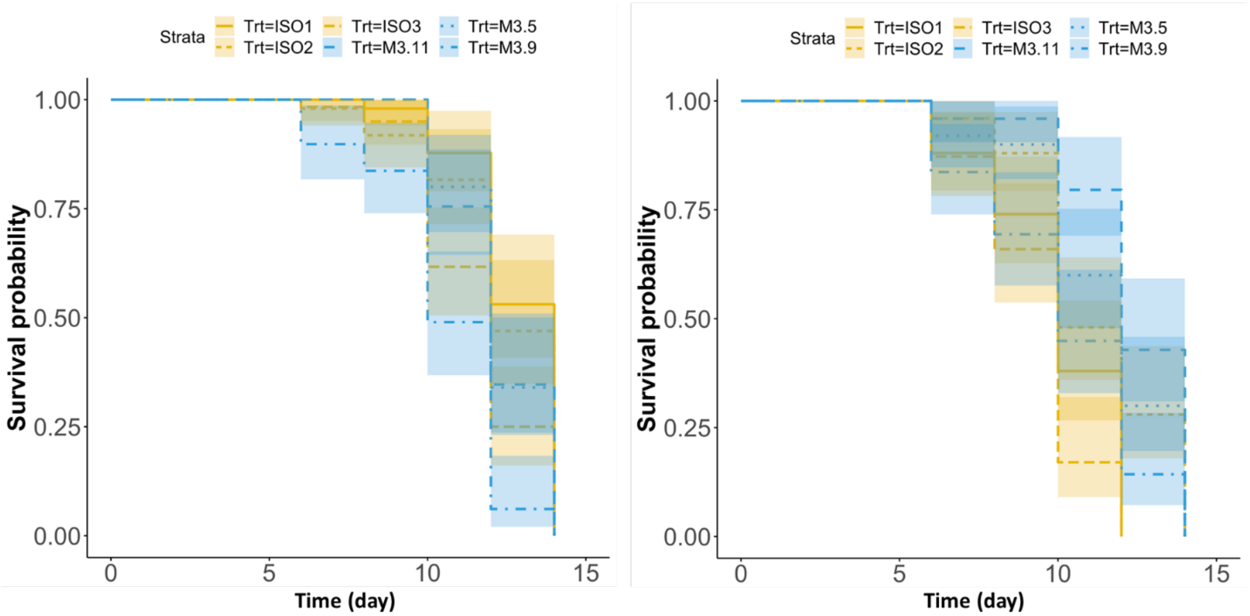

Figure S5. Knockout of the *mElo* orthologue *GI20347* in *D. mojavensis* did not lead to significant differences in survival at 37°C in a non-desiccating environment. Differences in survival between the wild type and *Dmoj/mElo* knockout strains of *D. mojavensis* were determined using the linear mixed effects model with the variation within each group (iso-female or independent knockout strains) being random effects. No significant differences were observed (Female: *P* = 0.4; Male: *P* = 0.2).

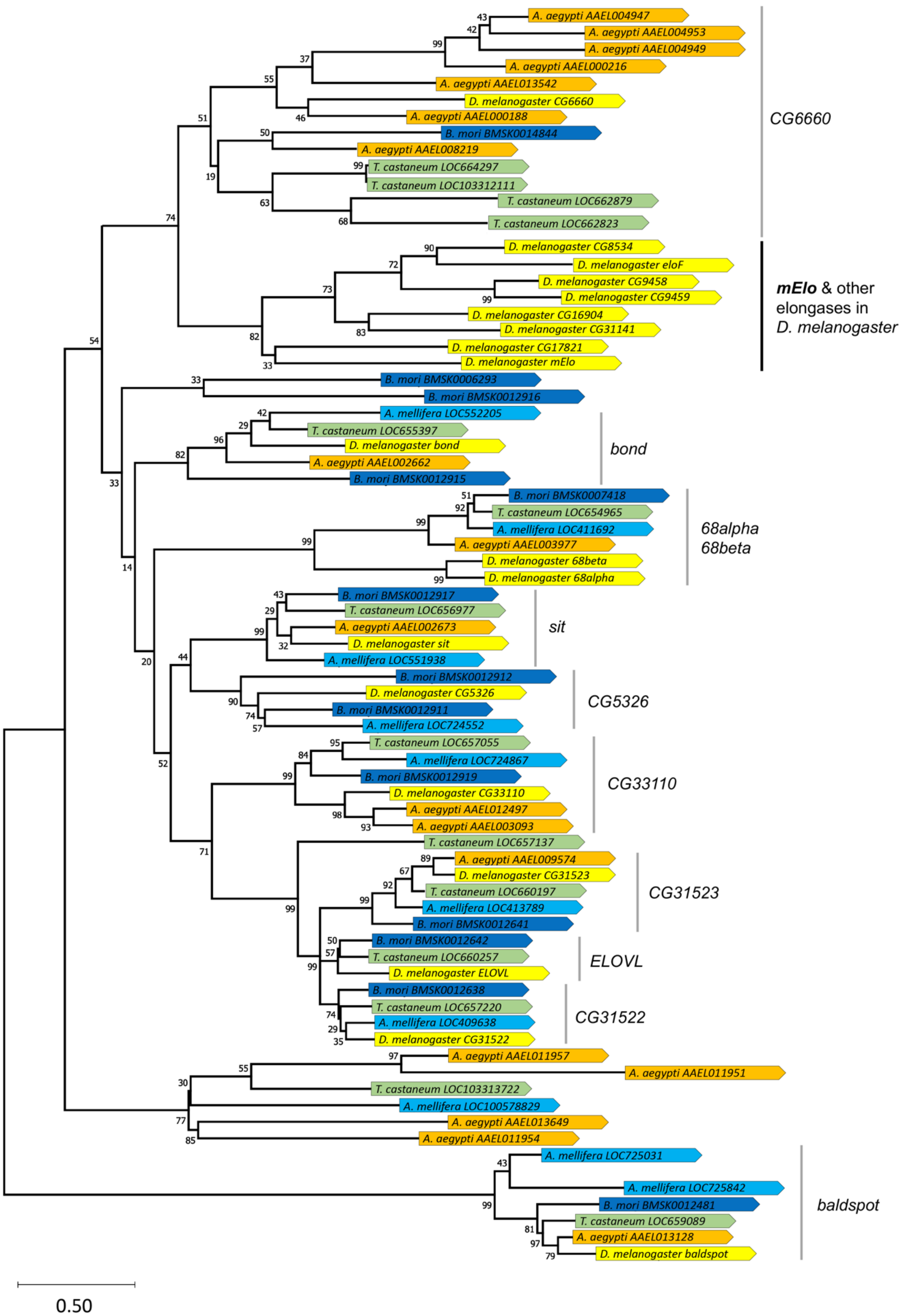
A. aegypti AAEL004947
A. aegypti AAEL004953
A. aegypti AAEL004949
A. aegypti AAEL000216
A. aegypti AAEL013542
D. melanogaster CG6660
A. aegypti AAEL000188
B. mori BMSK0014844
A. aegypti AAEL008219
T. castaneum LOC664297
T. castaneum LOC103312111
T. castaneum LOC662879
T. castaneum LOC662823
CG6660
D. melanogaster CG8534
D. melanogaster eloF
D. melanogaster CG9458
D. melanogaster CG9459
D. melanogaster CG16904
D. melanogaster CG31141
D. melanogaster CG17821
D. melanogaster mElo
mElo & other elongases in D. melanogaster
B. mori BMSK0006293
B. mori BMSK0012916
A. mellifera LOC552205
T. castaneum LOC655397
D. melanogaster bond
A. aegypti AAEL002662
B. mori BMSK0012915
bond
B. mori BMSK0007418
T. castaneum LOC654965
A. mellifera LOC411692
A. aegypti AAEL003977
D. melanogaster 68beta
D. melanogaster 68alpha
68alpha
68beta
B. mori BMSK0012917
T. castaneum LOC656977
A. aegypti AAEL002673
D. melanogaster sit
A. mellifera LOC551938
sit
B. mori BMSK0012912
D. melanogaster CG5326
B. mori BMSK0012911
A. mellifera LOC724552
CG5326
T. castaneum LOC657055
A. mellifera LOC724867
B. mori BMSK0012919
D. melanogaster CG33110
A. aegypti AAEL012497
A. aegypti AAEL003093
CG33110
T. castaneum LOC657137
A. aegypti AAEL009574
D. melanogaster CG31523
T. castaneum LOC660197
A. mellifera LOC413789
B. mori BMSK0012641
CG31523
B. mori BMSK0012642
T. castaneum LOC660257
D. melanogaster ELOVL
ELOVL
B. mori BMSK0012638
T. castaneum LOC657220
A. mellifera LOC409638
D. melanogaster CG31522
CG31522
A. aegypti AAEL011957
A. aegypti AAEL011951
T. castaneum LOC103313722
A. mellifera LOC100578829
A. aegypti AAEL013649
A. aegypti AAEL011954
A. mellifera LOC725031
A. mellifera LOC725842
B. mori BMSK0012481
T. castaneum LOC659089
A. aegypti AAEL013128
D. melanogaster baldspot
baldspot
0.50

Figure S6. Phylogenetic tree of all elongase genes identified in *Drosophila melanogaster*, *Aedes aegypti*, *Apis mellifera*, *Bombyx mori*, and *Tribolium castaneum*. The elongase genes of each species are denoted with a different color. The phylogenetic tree was inferred by the Maximum Likelihood method using amino acid sequences with 1000 bootstrap tests. The numbers next to nodes represent bootstrap values. The scale bar indicates the number of changes per site. The tree showed that three elongase genes, *bond*, *CG31523*, and *sit*, have one-to-one orthologs across all five species. *mElo* is clustered with a few *Drosophila* genes suggesting that this gene is likely to be *Drosophila* specific.

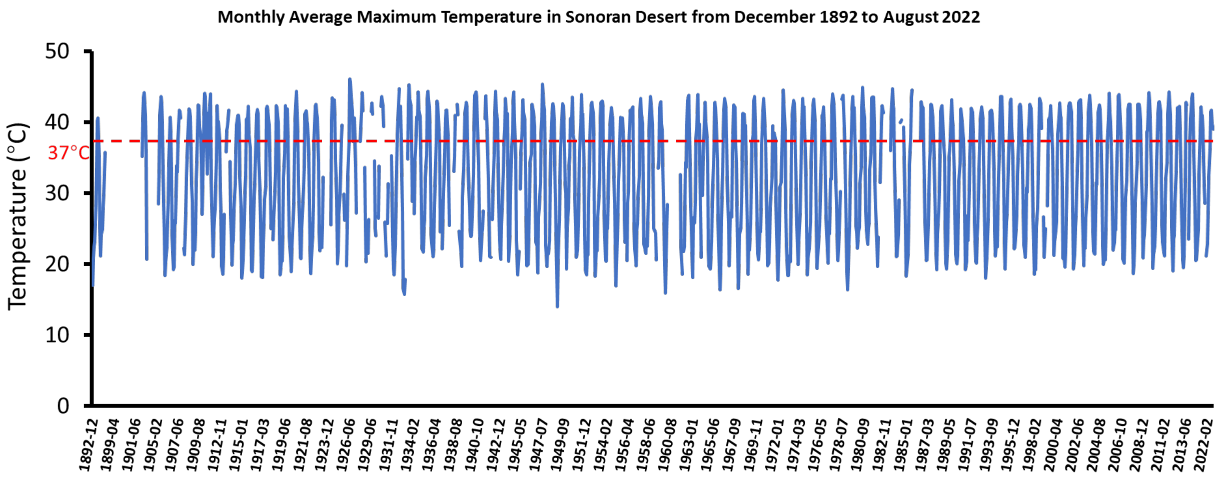

Figure S7. **Monthly average maximum temperatures in the Sonoran Desert**. The plot of monthly average maximum temperatures in a climatic station (GILA BEND 2 SE, AZ US) in the Sonoran Desert from December 1892 to August 2022. The climatic station is located at the coordinate 32.93803, -112.68109. The red dotted line indicates 37°C. The data were obtained from NCEI-NOAA (https://www.ncei.noaa.gov/).

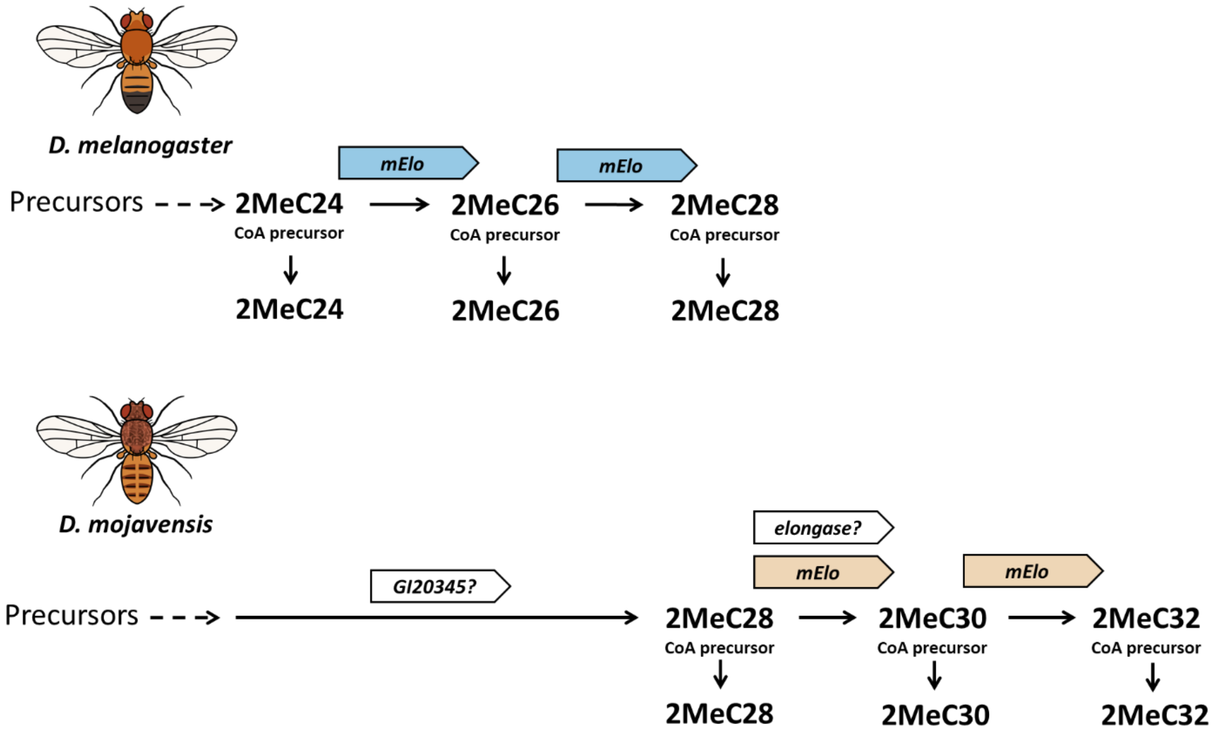

Figure S8. **Model showing the production of mbCHCs in *D. melanogaster* and *D. mojavensis*.** In *D. melanogaster*, the elongase *mElo* elongates 2MeC24 to 2MeC26 and 2MeC28. In *D. mojavensis*, the elongase *mElo* elongates 2MeC28 to 2MeC30 and 2MeC32, while elongation to 2MeC28 is due to another elongase, possibly *GI20345*, which is expressed in *D. mojavensis* oenocytes and can elongate shorter mbCHCs to 2MeC28 when overexpressed in *D. melanogaster* oenocytes. As the knockout of *mElo* did not fully reduce the production of 2MeC30 in *D. mojavensis*, we hypothesize that another elongase may also be involved in the synthesis of mbCHCs up to 2MeC30 in *D. mojavensis*.

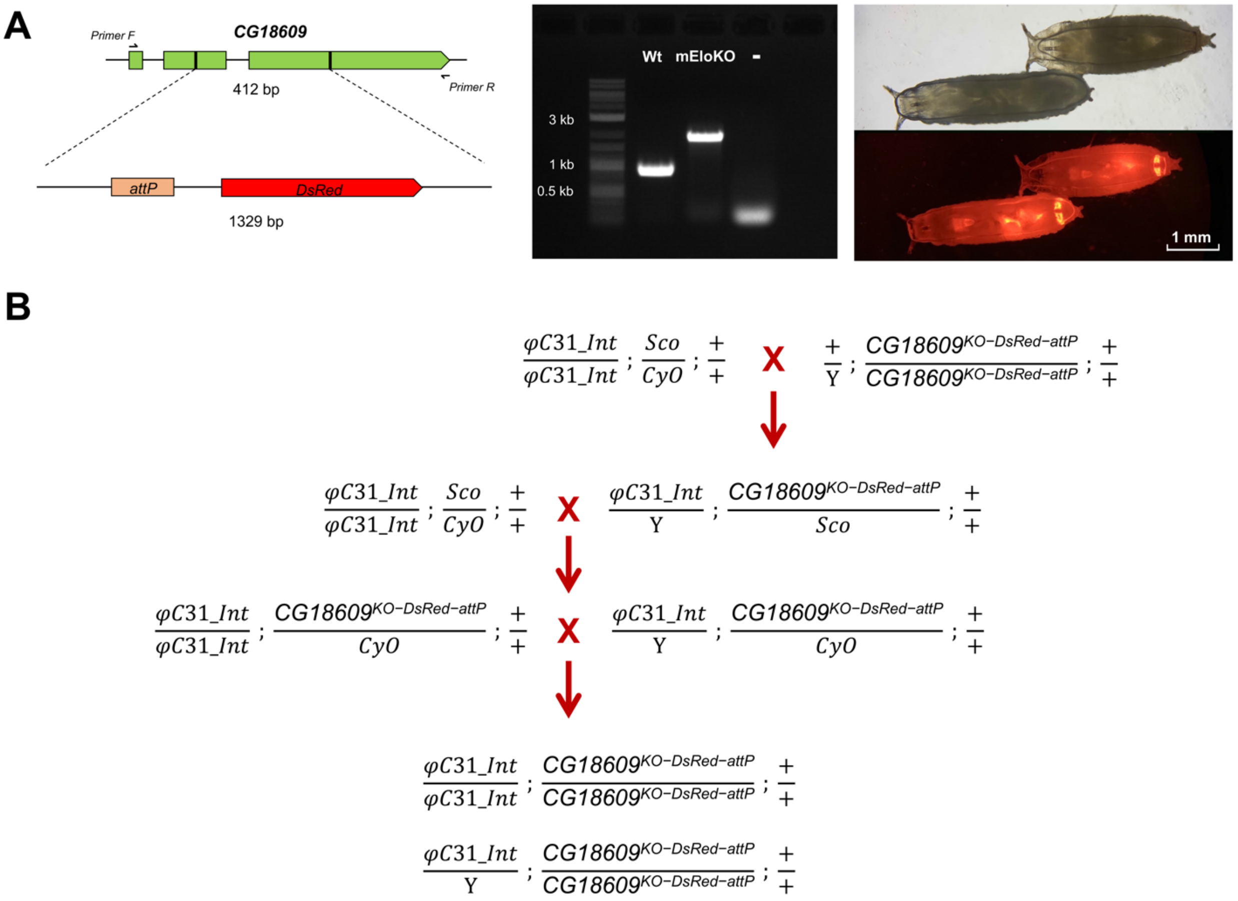

Figure S9. **Generation of *mElo* knockout lines in *D. melanogaster*.** A. Diagram of knockout using CRISPR/Cas9-mediated homology-directed repair on *Dmel/mElo* (Left panel). Successful knockout was confirmed with the replacement of the *attP*/*DsRed* sequence (Middle panel) as well as the presence of DsRed (Right panel). B. Crossing scheme to generate *w*^{1118}*, P{y*$^{+t7.7}$*=nos-phiC31\int.NLS}X; CG18609*$^{KO\text{-}DsRed\text{-}attP}$ (*mEloKOint)* strain.

Table S1. CHC profiles of female (top) and male (bottom) *D. melanogaster mEloKO* and *mElo* rescue strains. The abundance is in ng / fly. Student's *t*-test was used to determine the difference of each CHC between each transgenic line and the control. *P*-values were adjusted using Benjamini-Hochberg correction at *alpha* = 0.05. *N* =4-5. n.s.: not significant; *: $P < 0.05$; **: $P < 0.01$; ***: $P < 0.001$.

**Female_CHCs**	**Control**	***mEloKO***	***mElo* rescue**
**C23**	55.9 ± 8.5	76.1 ± 2.9 (n.s.)	289.7 ± 23.8 (***)
**7-C23:1**	60.5 ± 11.5	38.5 ± 1.8 (n.s.)	9.4 ± 5.8 (*)
**2MeC24**	25.2 ± 7.4	9.3 ± 1.4 (n.s.)	5.8 ± 0.5 (n.s.)
**C25 + 7-C25:1**	236.3 ± 14.6	304.2 ± 46.2 (n.s.)	353.3 ± 45.6 (n.s.)
**2MeC26**	206 ± 44.6	102.2 ± 6 (n.s.)	112.6 ± 11.3 (n.s.)
**C27**	57.7 ± 7.4	71.4 ± 6.6 (n.s.)	166.4 ± 26.4 (**)
**7,11-C27:2**	649.8 ± 92.6	430.8 ± 34.6 (n.s.)	227.2 ± 22.8 (*)
**2MeC28**	39 ± 7.4	1.7 ± 1 (*)	33.1 ± 2.6 (n.s.)
**7,11-C29:2**	198.6 ± 44	205.6 ± 16.7 (n.s.)	78.7 ± 19 (n.s.)

**Male_CHCs**	**Control**	***mEloKO***	***mElo* rescue**
**C21**	1.7 ± 1.7	23.7 ± 5.4 (*)	34.5 ± 7.1 (*)
**C22**	8.2 ± 3.2	29.1 ± 5.1 (*)	32.5 ± 6.5 (n.s.)
**C22:1**	7.4 ± 2.5	8 ± 4.7 (n.s.)	5.5 ± 3.3 (n.s.)
**C23**	137.7 ± 25.4	265 ± 20 (*)	281.1 ± 38.9 (n.s.)
**7-C23:1**	1049 ± 132	1138.4 ± 108.5 (n.s.)	1077.5 ± 130 (n.s.)
**5-C23:1**	58.6 ± 5.2	82.6 ± 7.4 (n.s.)	30.7 ± 17.7 (n.s.)
**2MeC24**	35.0 ± 8.3	38 ± 5.7 (n.s.)	27.8 ± 1.9 (n.s.)
**C25 + 7-C25:1**	87.2 ± 21.0	101.3 ± 17.5 (n.s.)	69.3 ± 8.1 (n.s.)
**2MeC26**	54.6 ± 9.2	14.7 ± 2.3 (*)	40.9 ± 2.6 (n.s.)
**C27**	1.6 ± 1.6	Trace (n.s.)	2.9 ± 1.7 (n.s.)
**2MeC28**	10.4 ± 3.2	Trace (*)	8.2 ± 0.4 (n.s.)

Table S2. CHC profiles for the female (top) and male (bottom) *OenoGAL4* > *UAS-O/E* of *CG18609* (*mElo*), *GI20343*, *GI20345*, and *GI20347* in the *attP40* background. The abundance is in ng / fly. Student's t-test was used to determine the difference of each CHC between each transgenic line and the control. *P*-values were adjusted using Benjamini-Hochberg correction at *alpha* = 0.05. *N* =6-7. n.s.: not significant; *: $P < 0.05$; **: $P < 0.01$; ***: $P < 0.001$.

**Female_CHCs**	**Control**	***CG18609***	***GI20343***	***GI20345***	***GI20347***
**C21**	Trace	Trace (n.s.)	6.6 ± 0.5 (***)	5.1 ± 1.6 (*)	Trace (n.s.)
**C22**	7 ± 0.4	7.3 ± 0.4 (n.s.)	Trace (***)	2.8 ± 1.8 (n.s.)	7.5 ± 0.7 (n.s.)
**C23**	179.6 ± 9.7	164 ± 7 (n.s.)	130.8 ± 9.7 (*)	144.3 ± 6.5 (*)	167.8 ± 15.6 (n.s.)
**7-C23:1**	67.9 ± 3.9	72.9 ± 5.2 (n.s.)	55.3 ± 9.4 (n.s.)	58.7 ± 6.1 (n.s.)	64.4 ± 4.1 (n.s.)
**2MeC24**	17.3 ± 1.8	10.9 ± 1.5 (n.s.)	22.9 ± 1.1 (*)	Trace (***)	3.2 ± 1.4 (***)
**C25 + 7-C25:1**	240.5 ± 30.1	173.1 ± 9.8 (n.s.)	187.0 ± 28.0 (n.s.)	219.2 ± 123.6 (n.s.)	198.2 ± 22.5 (n.s.)
**7,11-C25:2**	29.8 ± 2	30.7 ± 3.2 (n.s.)	14.1 ± 2.5 (**)	51 ± 7.4 (n.s.)	29.8 ± 2.4 (n.s.)
**2MeC26**	147.6 ± 3.8	147.6 ± 18.9 (n.s.)	129.4 ± 7.8 (n.s.)	95.7 ± 1.7 (***)	46.4 ± 13.6 (***)
**C27**	13.5 ± 1.3	29.7 ± 4.6 (*)	11.4 ± 4.3 (n.s.)	80.1 ± 6.5 (***)	48.4 ± 9.9 (*)
**7,11-C27:2**	449.3 ± 21.3	496.2 ± 44.3 (n.s.)	369.6 ± 60.2 (n.s.)	483.3 ± 55.1 (n.s.)	402.1 ± 26.8 (n.s.)
**2MeC28**	19.1 ± 1.2	44 ± 5.2 (**)	11.6 ± 1.2 (**)	44.8 ± 2.5 (***)	39.8 ± 8.5 (n.s.)
**7,11-C29:2**	94 ± 2.7	107.3 ± 7.9 (n.s.)	63.7 ± 9.2 (*)	158.1 ± 16.3 (**)	126.5 ± 13.6 (n.s.)
**2MeC30**	Trace	Trace (n.s.)	Trace (n.s.)	Trace (n.s.)	7 ± 0.7 (***)

**Male_CHCs**	**Control**	***CG18609***	***GI20343***	***GI20345***	***GI20347***
**C21**	11.2 ± 0.8	11.2 ± 1.5 (n.s.)	10.3 ± 1.3 (n.s.)	7.5 ± 0.7 (*)	5.4 ± 1.2 (**)
**C22**	22.9 ± 1.2	26.1 ± 2.7 (n.s.)	20.9 ± 1.9 (n.s.)	19 ± 1.4 (n.s.)	21.5 ± 1.3 (n.s.)
**C22:1**	9.1 ± 4.3	10 ± 0.7 (n.s.)	Trace (n.s.)	Trace (n.s.)	5.6 ± 1.2 (n.s.)
**C23**	286.9 ± 14.9	254.1 ± 5.8 (n.s.)	241.9 ± 25.2 (n.s.)	156.2 ± 8.4 (***)	283.4 ± 12.1 (n.s.)
**7-C23:1**	793.6 ± 30.6	799.8 ± 35.8 (n.s.)	795.1 ± 81.3 (n.s.)	322.4 ± 26.9 (***)	633.6 ± 14.1 (**)
**5-C23:1**	65.1 ± 2.2	63.3 ± 2.3 (n.s.)	53.2 ± 10.8 (n.s.)	39.7 ± 5.1 (**)	54.7 ± 1.3 (**)
**2MeC24**	13.4 ± 4.6	3.7 ± 2.3 (n.s.)	20.5 ± 1.7 (n.s.)	3.7 ± 1.8 (n.s.)	3.1 ± 1.4 (n.s.)
**C25 + 7-C25:1**	226.9 ±34.2	163.1 ± 9.7 (n.s.)	169.9 ± 26.7 (n.s.)	268.7 ± 28.0 (n.s.)	230.9 ± 11.4 (n.s.)
**2MeC26**	33.7 ± 2.3	37 ± 10.3 (n.s.)	60.3 ± 10.3 (n.s.)	28.6 ± 3.8 (n.s.)	12.2 ± 5.5 (*)

**C27**	12.8 ± 1.9	15.2 ± 1.4 (n.s.)	4.8 ± 2.2 (n.s.)	32.1 ± 6.2 (*)	14.6 ± 0.6 (n.s.)
**7-C27:1**	Trace	Trace (n.s.)	Trace (n.s.)	187.9 ± 45.6 (**)	Trace (n.s.)
**2MeC28**	13.6 ± 1.2	27.6 ± 6.7 (n.s.)	14.3 ± 3 (n.s.)	26.2 ± 3.1 (**)	20.7 ± 4.2 (n.s.)
**2MeC30**	Trace	Trace (n.s.)	Trace (n.s.)	Trace (n.s.)	6.9 ± 0.2 (***)

Table S3. CHC profiles for *D. mojavensis* with *mElo* knocked out in females (top) and males (bottom). ISO1, ISO2, and ISO3 are isofemale lines established from the parental population. M3.5, M3.9, and M3.11 are independent *mElo*-knockout lines with 5 bp insertion, 90 bp deletion, and 10 bp deletion on the third exon of *mElo*. The abundance is in ng / fly. $N = 6$. Linear mixed effects models were applied to determine the difference of each CHC between the wild type and knockout lines of *D. mojavensis*. The three isofemale wild type and independent knockout strains were included as random effects.

**Female_CHCs**	**ISO1**	**ISO2**	**ISO3**	**M3.5**	**M3.9**	**M3.11**	**Statistics**
2MeC28	7.9 ± 0.9	10 ± 1	7.8 ± 0.8	11.1 ± 0.6	9.5 ± 2.9	14.9 ± 4.7	$P = 0.1$
2MeC30	51.3 ± 6	53.1 ± 2.5	54.4 ± 5.2	22.5 ± 1.2	15.4 ± 3.1	23 ± 1.4	$t(4) = -11.6$, $P < 0.001$
2MeC32	7.7 ± 1.3	8.9 ± 1.3	5.9 ± 0.6	Trace	Trace	Trace	$t(4) = -8.5$, $P = 0.001$
C33:1	3.1 ± 1.3	Trace	1.3 ± 1.3	7.7 ± 0.9	2.2 ± 3.1	8.2 ± 3	$P = 0.09$
C35:2 (a)	25.3 ± 2.1	27.2 ± 1.8	28.3 ± 1.5	30.1 ± 2.5	22.5 ± 4.4	29.9 ± 8.3	$P = 0.8$
C35:2 (b)	5.9 ± 2.6	11.5 ± 3	16.2 ± 1.8	8.7 ± 0.5	1.2 ± 2.7	6.2 ± 5.9	$P = 0.2$
C37:2 (a)	12.7 ± 2	16.6 ± 1.6	17.4 ± 1.6	14 ± 0.9	7.7 ± 1.7	17.1 ± 3.4	$P = 0.4$
C37:2 (b)	15.1 ± 2.8	19.7 ± 2.5	27.8 ± 2.9	16.4 ± 1	9.4 ± 2.7	20.8 ± 5.6	$P = 0.3$

**Male_CHCs**	**ISO1**	**ISO2**	**ISO3**	**M3.5**	**M3.9**	**M3.11**	**Statistics**
2MeC28	8.6 ± 0.5	9.4 ± 0.9	8.2 ± 0.5	12 ± 1	8.9 ± 0.7	11 ± 2.5	$P = 0.1$
2MeC30	53.9 ± 2.7	62.3 ± 3.7	55.2 ± 3.6	24.3 ± 1.4	12.7 ± 2	21.9 ± 2	$t(4) = -8.5$, $P = 0.001$
2MeC32	6.3 ± 0.6	7.5 ± 0.7	6.5 ± 0.7	2.2 ± 1.4	Trace	Trace	$t(4) = -7.3$, $P = 0.002$
C33:1	3.8 ± 1.1	1.2 ± 1.2	Trace	6.2 ± 0.2	2.6 ± 3.8	7 ± 4.3	$P = 0.1$
C35:2 (a)	46.7 ± 2.8	54.1 ± 4.2	46.8 ± 2.2	45.3 ± 7.5	23.9 ± 3.8	42.4 ± 10.6	$P = 0.2$
C35:2 (b)	5.3 ± 3.4	Trace	12 ± 7.3	12.8 ± 5.5	4.2 ± 6.5	6.2 ± 8.8	$P = 0.7$
C37:2 (a)	9.5 ± 1	8.3 ± 1.3	9.1 ± 1.4	9.7 ± 2	5.5 ± 1	7 ± 1	$P = 0.3$
C37:2 (b)	15.8 ± 1.9	14.8 ± 1.9	12 ± 1.3	18.9 ± 4.2	7.5 ± 1.9	8.3 ± 2.3	$P = 0.5$

Table S4. Primers or Oligos used for this study

Name	Primers (5' to 3')
CG18609 ISH Probe F	cgccttcggaggtctcag
CG18609 ISH Probe R	ctcttcatcgtgggcatttgcaatc
GI20343 ISH Probe F	Tgcatggaatccttcccacttg
GI20343 ISH Probe R	gttgccgccgccaG
GI20344 ISH Probe F	GGGGTGTACCGAGTTATCATTAAT
GI20344 ISH Probe R	TTACTGCGTTTTCAATGTCTTGGG
GI20345 ISH Probe F	ATGGCTAGCTCCTTTCCACTG
GI20345 ISH Probe R	CTATTGAGTTTTCTTCGATTTTG
GI20347 ISH Probe F	GGCATTTGTGATCTGC
GI20347 ISH Probe R	CTTGATATAGAATTTGCCAAAC
Dmel/mElo-gRNA1-*BbsI*-F	CTTCGCAAGATCTTTATGAGAAAC
Dmel/mElo-gRNA1-*BbsI*-R	AAACGTTTCTCATAAAGATCTTGC
Dmel/mElo-gRNA2-*BbsI*-F	CTTCGGAGGCCATGTCAATGCCGT
Dmel/mElo-gRNA2-*BbsI*-R	AAACACGGCATTGACATGGCCTCC
Dmel/mElo-LeftHomo-*EcoRI*-F	gcGAATTCCCATGCTGTCCTCGGATCAT
Dmel/mElo-LeftHomo-*NotI*-R	gcGCGGCCGCTCTCATAAAGATCTTGCCCAATTT
Dmel/mElo-RightHomo-*AscI*-F	gcGGCGCGCCCGTTGGACTGCTGAACTCC
Dmel/mElo-RightHomo-*XhoI*-R	ggCTCGAGTTAGTACACACGGTTCTTTCCT
DmelCG18609-EcoRI-F	ggGAATTCATGCTCCGATACTTGCGCATAC
DmelCG18609-XbaI-R	ggTCTAGACTACGATTGCTTTGCGTTGATTTTCG
DmelCG17821-NdeI-F	ggCATATGATGAACTTCACACTATTGGATTTATTATT
DmelCG17821-XbaI-R	ggTCTAGATCACTGCTCTTTACTTTTGGCTTT
DmojGI20343-NdeI-F	ggCATATGATGCACGCATCGAATTCAAGTC
DmojGI20343-XbaI-R	ggTCTAGAATGCACGCATCGAATTCAAGTC
DmojGI20345-NdeI-F	ggCATATGATGGGCGTCGATATAATCGAAC
DmojGI20345-XbaI-R	ggTCTAGACTATTGAGTTTTCTTCGATTTTGGC
DmojGI20347-EcoRI-F	ggGAATTCATGCTCAATATTTTCAATATTC
DmojGI20347-XbaI-R	ggTCTAGATTACATTTGTTTGGAGCTCTTC
5'DmojGI20345-AscI-F	gcGGCGCGCCAATATTTCTTAAAATAAAAAAT
5'DmojGI20345-SbfI-R	ggCCTGCAGGTTTGCACTGCACTGTTTTCCAAAGCT
*Dmoj/white*_sgRNAa-F	GAAATTAATACGACTCACTATAggccagcagttc<br>gcccggatGTTTTAGAGCTAGAAATAGC
*Dmoj/white*_sgRNAb-F	GAAATTAATACGACTCACTATAGgatcaggagct<br>attgatacgGTTTTAGAGCTAGAAATAGC
Dmoj/mElo-sgRNAa-F	GAAATTAATACGACTCACTATAGgaggcaattga<br>agtgaccgcGTTTTAGAGCTAGAAATAGC
Dmoj/mElo-sgRNAb-F	GAAATTAATACGACTCACTATAGGcgcgtttacaatcttgg<br>ccGTTTTAGAGCTAGAAATAGC
sgRNA-CRISPR-R	AAAAGCACCGACTCGGTGCCACTTTTTCAAGTTGATAAC<br>GGACTAGCCTTATTTTAACTTGCTATTTCTAGCTCTAAAAC

Dataset S1. List of gene sequences that used in the phylogenetic analyses in **Figure 4** and **Figure S6**.
